# Phenolic Composition from Different Loquat (*Eriobotrya japonica* Lindl.) Cultivars Grown in China and Their Antioxidant Properties

**DOI:** 10.3390/molecules20010542

**Published:** 2015-01-05

**Authors:** Wenna Zhang, Xiaoyong Zhao, Chongde Sun, Xian Li, Kunsong Chen

**Affiliations:** 1 Laboratory of Fruit Quality Biology, Zhejiang University, Zijingang Campus, Hangzhou 310058, Zhejiang, China; E-Mails: nawen2007@163.com (W.Z.); zhaoxiaoyong-1989@163.com (X.Z.); adesun2006@zju.edu.cn (C.S.); akun@zju.edu.cn (K.C.); 2 The State Agriculture Ministry Laboratory of Horticultural Plant Growth, Development and Quality Improvement, Zhejiang University, Zijingang Campus, Hangzhou 310058, Zhejiang, China; 3 Zhejiang Key Laboratory for Agro-Food Processing, Zhejiang University, Zijingang Campus, Hangzhou 310058, Zhejiang, China

**Keywords:** *Eriobotrya japonica* Lindl., cultivars, phenolic compounds, antioxidant properties

## Abstract

China is one of the most important centers of diversity for *Eriobotrya japonica* Lindl. in the world. In this study, seven loquat cultivars grown in China were evaluated for their phenolic compounds and antioxidant activity. Eleven phenolic compounds, *i.e.*, 3-*p*-coumaroylquinincacid (3-*p*-CoQA), 5-caffeoylquinic acid (5-CQA), 4-caffeoylquinic acid (4-CQA), 3-caffeoylquinic acid (3-CQA), 5-feruloylquinic acid (5-FQA), quercetin-3-*O*-galactoside (Q-3-Gal), quercetin-3-*O*-glucoside (Q-3-Glu), quercetin-3-*O*-rhamnoside (Q-3-Rha), kaempferol-3-*O*-galactoside (K-3-Gal), kaempferol-3-*O*-rhamnoside (K-3-Rha), and kaempferol-3-*O*-glucoside (K-3-Glu) were identified and quantified in the peel and pulp of the cultivars tested. 3-CQA and 5-CQA were the predominant components in both fruit parts. 2,2-Diphenyl-1-picrylhydrazyl radicals (DPPH), 2,2'-azino-bis(3-ethylbenzothiazoline-6-sulfonate (ABTS), and ferric reducing antioxidant power (FRAP) assays were used for the antioxidant evaluation. Results showed that peel extracts had higher antioxidant activities than their pulp counterparts in all the cultivars tested, which was correlated with their higher total phenolic contents. The antioxidant potency composite (APC) index showed obvious variations ranging from 64.15 to 100 in the peel and from 59.49 to 97.95 in the pulp of different cultivars, where “Dahongpao” (DHP) and “Luoyangqing” (LYQ) had the highest APC index in the peel and pulp, respectively. Overall, loquat cultivars rich in hydroxycinnamic acids (HCAs) such as 3-*p*-CoQA, 5-CQA, 4-CQA, 3-CQA and 5-FQA showed relatively higher antioxidant activities, and may be excellent sources of phytochemicals and natural antioxidants.

## 1. Introduction

Epidemiological studies have shown that consumption of fruit and vegetables has great health benefits against chronic diseases, such as cardiovascular disease, cancer, and diabetes [1,2,3]. The health-promoting properties of fruit and vegetables are mainly due to the presence of various antioxidants, including phenolics [4].

Phenolic compounds are a large group of plant secondary metabolites. So far, more than 8000 dietary phenolics have been identified, and their distribution and accumulation profiles were affected by both genetic and environmental factors [3,4]. Loquat (*Eriobotrya japonica* Lindl.) is a subtropical evergreen perennial fruit tree originated in south-eastern China. It has been cultivated for more than 2000 years and is now commercially cultivated in more than 30 countries worldwide, including Japan, Turkey, Italy, Spain, Brazil, *etc.* Loquat fruit is delicious and is a good resource for dietary phenolics.

Loquat is a plant with high medicinal value since different organs have been used historically as folk medicines for thousands of years. Loquat extracts have been used for the treatment of cough, chronic bronchitis, inflammation, diabetes, and cancer in Chinese folk medicine, as recorded by ancient literature such as “*Compendium of Materia Medica*” [5]. The efficacy of loquat, as used in traditional Chinese medicine, is supported by current scientific evidence regarding the pharmacologically-active compounds in plant extracts and their structure-activity associations. For example, loquat extracts from leaf, flower, and kernel showed various pharmaceutical and health-promoting effects in different experimental models, such as antioxidant [6,7,8,9], anti-inflammation [10,11], anti-diabetic [12], anticancer [13], gastroprotective [14]. However, as the edible part, loquat fruit has been rarely investigated for their bioactive compounds and bioactivities.

China is the largest producer of loquat fruit in the world (170,000 ha). However, there is no extensive investigation of the phenolic profile and their antioxidant properties in loquat cultivars grown in China. The objective of the present study was to identify individual phenolic compounds in fruit peel and pulp of seven loquat cultivars and to evaluate their antioxidant properties. Results may useful for the selection of loquat genotypes rich in phenolic compounds and enhanced nutritional value, which may be important for better utilization of loquat genetic resources.

## 2. Results and Discussion

### 2.1. Fruit Quality Evaluation

All fruit used in the present research were harvested at the ready-to-eat stage. As shown in Table 1, fruit quality indices such as fruit weight (FW), fruit shape index (FSI), soluble solids content (SSC) varied significantly among the seven cultivars tested. The FW ranged from 24.24 (BZ) to 42.19 g (LYQ) among the cultivars tested. Previous studied showed that the FW of Turkey loquat cultivars ranged from 22.55 to 29.54 g [15], and those from Italy ranged from 38.4 to 74.2 g [16]. The cultivars tested showed similar fruit shape, as reflected by the similar FSI. SSC is an important fruit quality trait, which is closely related to consumer acceptance and satisfaction. In our study, the white-coloured cultivar NHB showed the lowest SSC (10.24 °Brix), while the red-coloured cultivar DHP showed the highest SSC (12.08 °Brix). Based on the literature, some Italian loquat cultivars showed higher SSC value up to 14.6 °Brix [16].

**Table 1:** Cultivars used in the present study and their basic fruit quality indices.

Cultivars	Abbreviation	Harvest Site (County, Province)	Colour	FW (g)	FSI	SSC (°Brix)
Baozhu	BZ	Tangxi, Zhejiang	Red	24.24 ± 3.56 ^e^	1.18 ± 0.10 ^a^	11.72 ± 1.74 ^ab^
Dahongpao	DHP	Tangxi, Zhejiang	Red	33.02 ± 6.66 ^c^	1.04 ± 0.09 ^b^	12.08 ± 0.89 ^a^
Dayeyangdun	DYYD	Tangxi, Zhejiang	Red	31.44 ± 3.47 ^cd^	1.08 ± 0.11 ^b^	12.02 ± 1.05 ^ab^
Jiajiao	JJ	Tangxi, Zhejiang	Red	40.01 ± 4.48 ^ab^	1.17 ± 0.08 ^a^	12.04 ± 1.02 ^ab^
Luoyangqing	LYQ	Luqiao, Zhejiang	Red	42.19 ± 1.28 ^a^	1.07 ± 0.05 ^b^	11.15 ± 0.73 ^bc^
Ninghaibai	NHB	Ninghai, Zhejiang	White	28.60 ± 3.79 ^d^	1.10 ± 0.06 ^ab^	10.24 ± 1.20 ^c^
Ruantiaobaisha	RTBS	Luqiao, Zhejiang	White	37.52 ± 1.00 ^b^	1.05 ± 0.05 ^b^	11.25 ± 1.70 ^b^

Abbreviation: FW, fruit weight; FSI, fruit shape index; SSC, soluble solid content; Data are expressed as mean ± standard deviation of twelve samples; Different superscripts within columns represent significant differences at *p* < 0.05.

### 2.2. Total Phenolic Contents

**Figure 1:**
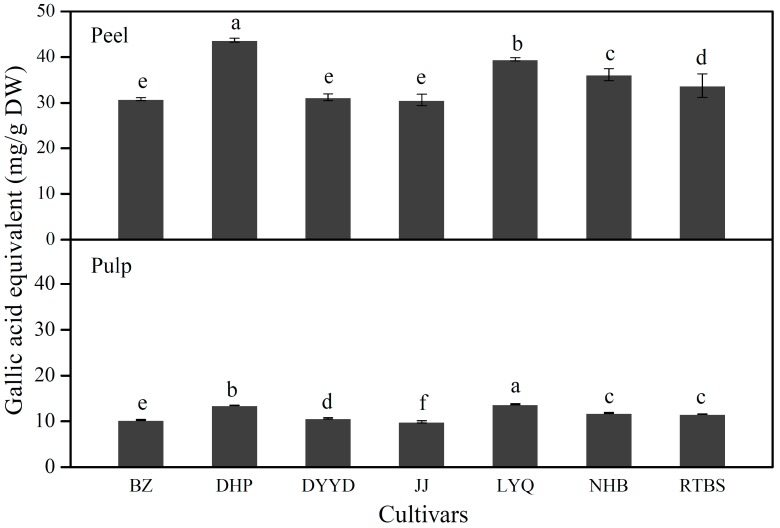
Total phenolic contents of the peel and pulp extracts of the seven loquat cultivars. Data are expressed as mean ± standard deviation of triplicate samples; Different letters within the same fruit tissue (peel or pulp) represent significant differences at *p* < 0.05; Cultivar abbreviations were shown in Table 1.

Due to the significant correlations found between the phenolic contents and various bioactivities including antioxidant activity, numerous studies have been carried out to select new genotypes rich in phenolic compounds [17,18]. Total phenolic contents of the peel and pulp extracts of seven loquat cultivars were measured using a modified colorimetric Folin-Ciocalteu method [19]. Total phenolic contents showed obvious variations among the cultivars tested, ranging from 30.58 (JJ) to 43.70 mg gallic acid equivalent (GAE)/g DW (DHP) for the peel and from 9.90 (JJ) to 13.73 mg GAE/g DW (LYQ) for the pulp (Figure 1). Total phenolic contents have been evaluated for loquat cultivars in China [20,21], Japan [17], Turkey [22,23] and America [24]. The variation in total phenolic contents of loquat fruit is due to both the genetic and environmental factors.

### 2.3. Identification of Individual Phenolic Compounds

Identification of individual phenolic compounds in loquat fruit were further carried out by HPLC-DAD and LC-ESI-MS/MS. For the identification of hydroxycinnamic acids (HCAs), the fragment ion information from LC-MS/MS were compared with the study of Clifford *et al*. [25]. As a result, five HCAs were identified in loquat fruit (Table 2). Among them, the [M−H]^−^ ion at *m/z* 336.9 indicated a molecular weight of 338 and [*p*-coumaric−H]^−^ ion at *m/z* 163.1 indicated the compound as *p*-coumaroylquininc acid (*p*-CoQA). Three caffeoylquinic acids showed the same [M−H]^−^ ion at *m/z* 353.0 indicating the same molecular weight of 354, and they all showed the [quinic-H]^−^ ion at *m/z* 191.0 or 190.8 and either a [caffic−H]^−^ ion at *m/z* 179.1 or [caffic−H−CO_2_]^−^ ion at *m/z* 135.1. The [M−H]^−^ ion at *m/z* 367.0 indicated a molecular weight of 368 and [quinic−H]^−^ ion at *m/z* 191.0 indicated compound as feruloylquinic acid (FAQ). Further linkage position of *p*-coumaroyl, caffic, and feruloyl residues on the quinic acid were analyzed according to the rules reported by Clifford *et al.* [25], and together with the confirmation of chemical standards, five HCAs were identified as 3-*p*-CoQA, 5-CQA, 4-CQA, 3-CQA, and 5-FQA, respectively.

For the identification of flavonols, the fragment ion information from LC-MS/MS were compared with the study of Hvattum *et al.* [26]. As a result, six flavonols were identified (Table 2). Among them, three quercetin glucosides showed [M−H]^−^ ions at *m/z* 463.3 or 447.2, which indicated a molecular weight of 464 or 448, and they all showed a [quercetin−H]^−^ ion at *m/z* 301.1 or 301.2 and a [quercetin−2H]^−^ ion at *m/z* 300.1 or 300.3. Three kaempferol glucosides showed [M−H]^−^ ion at *m/z* 447.2 or 431.3 indicated the molecular weight of 448 or 432, and they all showed a [kaempferol−H]^−^ ion at *m/z* 285.0 or 285.2 and a [kaempferol−2H]^−^ ion at *m/z* 284.2 or 284.1. Further glucosides type and linkage position of glucosides on the quercetin or kaempferol were analyzed according to the rule reported by Hvattum *et al.* [26], and together with the confirmation of chemical standards, six flavonols were identified as Q-3-Gal, Q-3-Glu, Q-3-Rha, K-3-Gal, K-3-Rha and K-3-Glu, respectively. By comparison with previous report [18], K-3-Gal and K-3-Rha identified in the present study were reported in loquat fruit for the first time.

**Table 2:** Structural and chromatographic characteristics of investigated phenolic compounds.

Structural Formula	Compounds	R_1_	R_2_	λ_max_ (nm)	Molecular Weight	ESI-MS^2^ (m/s)
HCAs 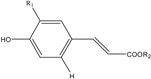	3*-p*-CoQA	H	3-quinic acid	226.1, 310.3	338	336.9, 163.1, 119.0
	5-CQA	OH	5-quinic acid	241.4, 324.6	354	353.0, 191.0, 135.1
	4-CQA	OH	4-quinic acid	240.2, 327.0	354	353.0, 190.8, 179.1
	3-CQA	OH	3-quinic acid	241.4, 324.6	354	353.0, 191.0, 135.1
	5-FQA	OCH_3_	5-quinic acid	216.6, 325.8	368	367.0, 191.0, 85.0
Flavonols 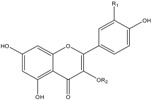	Q-3-Gal	OH	galactoside	255.6, 352.8	464	463.3, 301.1, 300.1
	Q-3-Glu	OH	glucoside	255.6, 352.8	464	463.3, 301.2, 300.1
	Q-3-Rha	OH	rhamnoside	255.6, 348.0	448	447.2, 301.2, 300.3
	K-3-Gal	H	galactoside	265.1, 346.8	448	447.2, 285.0, 284.2
	K-3-Rha	H	rhamnoside	263.9, 341.1	432	431.3, 285.2, 284.2
	K-3-Glu	H	glucoside	253.9, 349.2	448	447.2, 285.2, 284.1

### 2.4. Quantification of Individual Phenolic Compounds

Loquat peel extracts contained relatively higher amounts of phenolics than their pulp counterparts. In addition, both HCAs and Flavonols can be detected in the peel extracts, while only HCAs were detected in the pulp extracts. Both 5-CQA and 3-CQA were the main phenolic compounds in both fruit parts (Table 3 and Table 4).

For HCAs in both fruit peel and pulp, the contents of 3-CQA were relatively higher than those of 5-CQA. The contents of 3-CQA varied from 3.13 (JJ) to 6.75 mg/g DW (NHB) in the peel, and from 2.65 (JJ) to 6.29 mg/g DW (LYQ) in the pulp. The contents of 5-CQA varied from 1.92 (JJ) to 5.10 mg/g DW (LYQ) in the peel, and from 0.46 (BZ) to 1.42 mg/g DW (LYQ) in the pulp. The contents of 3-*p*-CoQA, 4-CQA, and 5-FQA were much lower compared with those of 3-CQA and 5-CQA. In both the fruit parts, 3-*p*-CoQA varied from ND to 0.52 mg/g DW, 4-CQA varied from 0.02 to 0.55 mg/g DW, and 5-FQA varied from 0.13 to 0.98 mg/g DW. For the total individual hydroxycinnamic acids (TIHCAs), *i.e*., the sum of five individual hydroxycinnamic acids identified in the present study, the cultivar NHB and LYQ showed the highest content in the peel (12.8 mg/g DW) and pulp (8.08 mg/g DW), respectively, while JJ showed the lest contents both in the peel (5.66 mg/g DW) and in the pulp (3.49 mg/g DW) (Table 3 and Table 4).

For the six flavonols detected in the fruit peel, DHP showed the highest contents of all three quercetin glucosides (Q-3-Gal, Q-3-Glu, and Q-3-Rha), while JJ contained the highest contents of all three kaempferol glucosides (K-3-Gal, K-3-Rha, and K-3-Glu). For the total individual flavonols (TIFs), *i.e*., the sum of six individual flavonols identified in the present study, it varied from 0.39 (LYQ) to 1.74 mg/g DW (JJ) in the peel of seven loquat cultivars (Table 3).

**Table 3:** Contents of individual phenolic compounds in the peel of seven loquat cultivars (mg/g DW).

Compounds		Cultivars						
		BZ	DHP	DYYD	JJ	LYQ	NHB	RTBS
HCAs	3-p-CoQA	0.13 ± 0.01 ^e^	0.41 ± 0.01 ^b^	0.36 ± 0.01 ^c^	0.07 ± 0.002 ^f^	0.52 ± 0.01 ^a^	0.39 ± 0.02 ^b^	0.27 ± 0.003 ^d^
	5-CQA	2.06 ± 0.10 ^d^	4.60 ± 0.05 ^b^	4.28 ± 0.02 ^c^	1.92 ± 0.01 ^e^	5.10 ± 0.10 ^a^	4.61 ± 0.08 ^b^	4.25 ± 0.07 ^c^
	4-CQA	0.16 ± 0.01 ^e^	0.36 ± 0.001 ^c^	0.21 ± 0.002 ^d^	0.12 ± 0.01 ^f^	0.37 ± 0.01 ^c^	0.55 ± 0.01 ^a^	0.40 ± 0.001 ^b^
	3-CQA	4.74 ± 0.18 ^e^	5.25 ± 0.04 ^c^	5.01 ± 0.12 ^d^	3.13 ± 0.08 ^f^	4.71 ± 0.09 ^e^	6.75 ± 0.11 ^a^	6.36 ± 0.10 ^b^
	5-FQA	0.45 ± 0.02 ^e^	0.98 ± 0.02 ^a^	0.21 ± 0.01 ^g^	0.42 ± 0.01 ^f^	0.82 ± 0.02 ^b^	0.50 ± 0.01 ^d^	0.73 ± 0.004 ^c^
Flavonols	Q-3-Gal	0.34 ± 0.01 ^b^	0.51 ± 0.01 ^a^	0.32 ± 0.01 ^c^	0.30 ± 0.01 ^d^	0.09 ± 0.01 ^g^	0.23 ± 0.004 ^e^	0.18 ± 0.004 ^f^
	Q-3-Glu	0.15 ± 0.01 ^b^	0.19 ± 0.004 ^a^	0.13 ± 0.004 ^d^	0.14 ± 0.004 ^c^	0.03 ± 0.003 ^g^	0.09 ± 0.003 ^e^	0.06 ± 0.001 ^f^
	Q-3-Rha	0.49 ± 0.02 ^b^	0.58 ± 0.01 ^a^	0.50 ± 0.02 ^b^	0.46 ± 0.002 ^c^	0.15 ±0.02 ^f^	0.42 ± 0.01 ^d^	0.35 ± 0.01 ^e^
	K-3-Gal	0.13 ± 0.003 ^d^	0.16 ± 0.004 ^b^	0.15 ± 0.003 ^c^	0.21 ± 0.004 ^a^	0.05 ± 0.002 ^e^	0.05 ± 0.001 ^e^	ND
	K-3-Rha	0.34 ± 0.01 ^c^	0.22 ± 0.002 ^d^	0.48 ± 0.01 ^b^	0.52 ± 0.01 ^a^	0.06 ± 0.001 ^f^	0.08 ± 0.001 ^e^	0.07 ± 0.001 ^e^
	K-3-Glu	0.08 ± 0.003 ^b^	0.01 ± 0.003 ^b^	0.08 ± 0.01 ^b^	0.11 ± 0.002 ^a^	ND	ND	ND
TIHCAs		7.52 ± 0.30 ^e^	11.60 ± 0.09 ^c^	10.06 ± 0.13 ^d^	5.66 ± 0.10 ^f^	11.53 ± 0.23 ^c^	12.80 ± 0.23 ^a^	12.02 ± 0.16 ^b^
TIFs		1.54 ± 0.05 ^c^	1.73 ± 0.02 ^a^	1.65 ± 0.05 ^b^	1.74 ± 0.01 ^a^	0.39 ± 0.01 ^f^	0.86 ± 0.02 ^d^	0.67 ± 0.01 ^e^

ND, not detected; Abbreviation: TIHCAs, total individual hydroxycinnamic acids; TIFs, total individual flavonols; Data are expressed as mean ± standard deviation of triplicate samples; Different superscripts between rows represent significant differences between samples (*p* < 0.05); Except 3-*p*-CoQA was quantified with *p*-coumaric acid, 5-FQA was quantified with ferulic acid, other phenolics were quantified with their own standard curves (mg/g DW).

**Table 4:** Contends of individual phenolic compounds in the pulp of seven loquat cultivars (mg/g DW).

Compounds		Cultivars						
		BZ	DHP	DYYD	JJ	LYQ	NHB	RTBS
HCAs	3-p-CoQA	ND	ND	0.06 ± 0.002 ^a^	0.02 ± 0.000 ^b^	ND	ND	ND
	5-CQA	0.46 ± 0.02 ^e^	0.76 ± 0.01 ^c^	0.87 ± 0.01 ^b^	0.57 ± 0.01 ^d^	1.42 ± 0.11 ^a^	0.80 ± 0.02 ^bc^	0.82 ± 0.04 ^bc^
	4-CQA	0.02 ± 0.002 ^c^	0.05 ± 0.01 ^b^	0.05 ± 0.001 ^b^	0.02 ± 0.001 ^c^	0.09 ± 0.01 ^a^	0.04 ± 0.01 ^b^	0.05 ± 0.003 ^b^
	3-CQA	3.88 ± 0.15 ^c^	6.09 ± 0.02 ^a^	3.47 ± 0.04 ^d^	2.65 ± 0.06 ^e^	6.29 ± 0.45 ^a^	4.49 ± 0.07 ^b^	4.75 ± 0.23 ^b^
	5-FQA	0.20 ± 0.004 ^d^	0.24 ± 0.01 ^b^	0.13 ± 0.002 ^f^	0.23 ± 0.01 ^bc^	0.29 ± 0.03 ^a^	0.17 ± 0.01 ^e^	0.21 ± 0.01 ^cd^
TIHCAs		4.56 ± 0.17 ^d^	7.13 ± 0.02 ^b^	4.58 ± 0.06 ^d^	3.49 ± 0.08 ^e^	8.08 ± 0.59 ^a^	5.50 ± 0.11 ^c^	5.83 ± 0.26 ^c^

ND, not detected; Abbreviation: TIHCAs, total individual hydroxycinnamic acids; Data are expressed as mean ± standard deviation of triplicate samples; Different superscripts between rows represent significant differences between samples (*p* < 0.05); Except 3-*p*-CoQA was quantified with *p*-coumaric acid, 5-FQA was quantified with ferulic acid, other phenolics were quantified with their own standard curves (mg/g DW).

### 2.5. Antioxidant Activity

The antioxidant activities of loquat cultivars were evaluated by DPPH, ABTS and FRAP methods. Generally speaking, these three assays showed consistent results for both the peel and pulp extracts of seven loquat cultivars (Table 5).

**Table 5:** Antioxidant capacities of peel and pulp extracts of seven loquat cultivars (mg TEAC/g DW).

Tissues	Cultivars	DPPH	ABTS	FRAP	APC Index	Rank
Peel	BZ	25.98 ± 0.97 ^e^	37.10 ± 1.67 ^d^	37.21 ± 0.33 ^f^	65.98	6
	DHP	36.64 ± 0.88 ^a^	57.32 ± 1.31 ^a^	59.71 ± 1.10 ^a^	100	1
	DYYD	26.25 ± 0.62 ^e^	36.19 ± 1.47 ^d^	42.08 ± 0.55 ^e^	68.42	5
	JJ	25.19 ± 0.61 ^e^	36.11 ± 0.56 ^d^	36.25 ± 0.98 ^f^	64.15	7
	LYQ	33.79 ± 0.27 ^b^	52.20 ± 1.63 ^b^	53.96 ± 0.93 ^b^	91.22	2
	NHB	30.72 ± 0.67 ^c^	43.76 ± 3.01 ^c^	48.68 ± 1.70 ^c^	80.56	3
	RTBS	29.34 ± 0.70 ^d^	42.65 ± 0.92 ^c^	44.83 ± 0.43 ^d^	76.52	4
Pulp	BZ	6.62 ± 0.51 ^d^	7.30 ± 0.27 ^d^	11.59 ± 0.66 ^d^	59.49	7
	DHP	11.79 ± 1.52 ^a^	11.68 ± 0.31 ^b^	17.73 ± 0.18 ^a^	97.03	2
	DYYD	7.23 ± 0.34 ^cd^	7.76 ± 0.29 ^d^	12.24 ± 0.44 ^c^	63.60	5
	JJ	7.11 ± 0.85 ^cd^	7.47 ± 0.56 ^d^	10.65 ± 0.23 ^e^	59.53	6
	LYQ	11.06 ± 0.87 ^a^	12.77 ± 0.34 ^a^	17.79 ± 0.18 ^a^	97.95	1
	NHB	8.91 ± 0.48 ^bc^	9.54 ± 0.11 ^c^	13.87 ± 0.15 ^b^	76.08	3
	RTBS	9.10 ± 1.18 ^b^	9.19 ± 0.77 ^c^	13.89 ± 0.68 ^b^	75.74	4

Data were expressed as mean ± standard deviation of triplicate samples; Different superscripts within the same column for each fruit parts (peel or pulp) represent significant differences between samples (*p* < 0.05); Antioxidant capacities (DPPH, FRAP, and ABTS) were calculated as mg TEAC/g DW.

DPPH values of the different cultivars analysed varied from 25.19 to 36.64 mg trolox equivalent antioxidant capacity (TEAC)/g DW in the peel and from 6.62 to 11.79 mg TEAC/g DW in the pulp (Table 5). DHP showed the highest DPPH values in both the peel and pulp tissues, followed by LYQ. And much higher levels of DPPH• radical scavenging activity were found in the peel fraction when compared with their pulp counterparts.

The ABTS values of the different cultivars analyzed varied from 36.11 to 57.32 mg TEAC/g DW in the peel and from 7.30 to 12.77 mg TEAC/g DW in the pulp (Table 5). DHP and LYQ showed the highest ABTS values in the peel and pulp tissues, respectively. There much higher levels of ABTS^+^ radical scavenging activity were found in the peel fraction when compared with their pulp counterparts.

The FRAP values of the loquat cultivars varied from 36.25 to 59.71 mg TEAC/g DW in the peel and from 10.65 to 17.79 mg TEAC/g DW in the pulp. The peel of DHP and pulp of LYQ showed the highest FRAP values among all the samples tested (Table 5). Similarly, higher FRAP values were found for extracts from the peel compared to the pulp, which was in accord with the results obtained by Pande *et al.* [24].

Previous studies also showed the antioxidant activities of loquat fruit grown in different regions, and values of DPPH, ABTS and FRAP values varied from 1.45 to 5.85 μmol TEAC/g FW, from 1.32 to 4.53 μmol TEAC/g FW, and from 2.14 to 5.91 μmol TEAC/g FW, respectively [21,22]. Such variations were results of different chemical compositions, which was affected by variety, stage of maturity and cultivation environment.

For a comprehensive comparison of the antioxidant capacities in two fruit parts of loquat of different cultivars, an overall antioxidant potency composite (APC) index was calculated according to the method described by Seeram *et al.* [27]. The APC index showed obvious variations ranging from 64.15 (JJ) to 100 (DHP) in the peel and from 59.49 (BZ) to 97.95 (LYQ) in the pulp (Table 5). Both DHP and LYQ are the main loquat cultivar in the market, and the high APC values for both these two cultivars indicated they may also have better health promoting values than other cultivars.

### 2.6. Correlations Analysis

Correlation analysis was carried between the antioxidant capacities and the phenolic contents in different loquat samples (Table 6). Significant correlations between DPPH, ABTS and FRAP were observed, providing validation of these three antioxidant activity evaluation methods, as mentioned above. Total phenolics showed strong correlation with antioxidant activities, indicating thatextracts with higher total phenolics showed higher antioxidant activity, and *vice versa*. Such data was in accord with previous results [21,22]. In addition, TIHCAs also showed high correlation with the antioxidant activities in loquat fruit, with higher correlation coefficient in the pulp (r ranged from 0.851 to 0.959, *p* < 0.01). High antioxidant activities of HCAs such as *p*-hydroxycinnamic acids were reported previously [28,29]. The high correlation between TIHCAs and three antioxidant activities indicated that HCAs may contribute significantly to the antioxidant activities of loquat fruit samples. TIFs, however, did not show a good correlation with the antioxidant activities. This may mainly due to their low contents in the loquat fruit samples.

**Table 6:** Correlation coefficients between phenolic content and antioxidant capacities.

Antioxidant Capacities/Phenolic Content	Peel			Pulp		
	DPPH	ABTS	FRAP	DPPH	ABTS	FRAP
DPPH	1	0.974 **	0.975 **	1	0.893 **	0.907 **
ABTS	0.974 **	1	0.956 **	0.893 **	1	0.963 **
Total phenolics	0.972 **	0.968 **	0.961 **	0.905 **	0.977 **	0.993 **
TIHCAs	0.696 **	0.616 **	0.753 **	0.851 **	0.931 **	0.959 **
TIFs	−0.294	−0.267	−0.279	−	−	−

** represents statistical significance at *p* < 0.01; −: no TIF was detected in loquat pulp.

## 3. Experimental Section

### 3.1. Chemicals

The chemical standards of *p*-coumaric acid, 5-CQA, 4-CQA, 3-CQA, ferulic acid, Q-3-Gal, Q-3-Glu, Q-3-Rha, K-3-Glu, Folin-Ciocalteu reagent (2 mol/L), Trolox, DPPH•, ABTS^+^, 2,4,6-tris(2-pyridyl)-s-triazine (TPTZ), and acetonitrile of chromatographic grade were purchased from Sigma-Aldrich (St. Louis, MO, USA). The chemical standards of K-3-Gal and K-3-Rha were purchased from Tauto Biotechnique (Shanghai, China). Double-distilled water (ddH_2_O) was used in all experiments and samples for HPLC were filtered through a 0.22 μm membrane before injection. All the other reagents of analytical grade were bought from Sinopharm Chemical Reagent Co., Ltd (Shanghai, China).

### 3.2. Materials

Seven loquat cultivars were harvested at optimum maturity on the basis of uniformity of shape and colour, absence of disease and mechanical damage in Zhejiang Province, China in the fruit season of 2013 (Table 1). In detail, LYQ and RTBS were harvested from Luqiao county; BZ, DHP, DYYD, and JJ from Tangxi county; NHB from Ninghai county. During the experiments, 90 fruits per cultivar, 30 for each of three replicates were kept separately and the fruit were separated into two parts, *i.e*., peel and pulp, and frozen in liquid nitrogen. After freeze-drying (FM 25EL-85, VirTis, Los Angeles, CA, USA), all the samples were ground into fine powder and stored at −80 °C until extraction and analysis of phenolics.

### 3.3. Fruit Quality Analysis

Twelve fruits of each cultivar were randomly chosen and quality traits such as FW, FSI, SSC were measured. Pulp colour was recorded as white and red. The height and diameter at the widest point of the fruit were measured with a vernier caliper, and the height/diameter ratio was calculated for FSI. SSC was measured with a digital refractometer (Atago PR-101R, Tokyo, Japan), and data was expressed as °Brix.

### 3.4. Preparation of Fruit Peel and Pulp Extracts

The ground powder of peel (0.15 g) and pulp (0.30 g) were extracted with 95% aqueous ethanol with 1% formic acid (7 mL) by sonication for 30 min. The ultrasonic frequency and power were 60 kHz and 30 W, respectively. The extracts were centrifuged at 12,879 *g* for 10 min at 4 °C and the residue was extracted twice as above. Both supernatants were combined and used for the determination of phenolic compounds and antioxidant activity.

### 3.5. Determination of Total Phenolics

Total phenolics of fruit extracts were measured using a modified colorimetric Folin-Ciocalteu method [19]. Four milliliters of ddH_2_O and appropriately diluted fruit extracts (0.5 mL) were placed in a test tube. Folin-Ciocalteu reagent (0.5 mol/L, 0.5 mL) was added to the solution and allowed to react for 3 min. The reaction was neutralized with saturated sodium carbonate (1 mL). Absorbance at 760 nm was measured using a spectrophotometer (UV-2550, Shimadzu, Tokyo, Japan) after 2 h. Standard solutions of gallic acid at concentrations of 0.1, 0.2, 0.3, 0.4, 0.5, and 0.6 mg/mL were used to plot the standard curve. Data were expressed as mg GAE/g DW.

### 3.6. HPLC-DAD and LC-ESI-MS/MS Analysis of Phenolic Compounds

Each individual phenolic compound in the fruit extracts was identified by LC-ESI-MS/MS. HCAs were detected at 280 nm; flavonols at 350 nm. Except 3-*p*-CoQA and 5-FQA were quantified with *p*-coumaric acid and ferulic acid, other phenolics were quantified with their own standard curves, and data were expressed as mg/g DW.

Individual phenolic compounds were firstly analyzed by HPLC (2695 pump, 2996 diode array detector, Waters, Milford, MA, USA) coupled with an ODS C18 analytical column (4.6 × 250 mm). The flow rate was 1 mL/min, the column temperature 25 °C, the injection volume 10 µL. The compounds were detected between 200 and 550 nm. The mobile phase of HPLC consisted of 0.1% (v/v) formic acid in water (eluent A) and 0.1% formic acid in acetonitrile (eluent B). The gradient program was as follows: 0 min, 5% B; 50 min, 28% B; 60 min, 43% B; 65 min, 43% B; 70 min, 5% B; 75 min, 5% B.

Mass spectrometric analyses were performed by an Agilent 6460 triple quadrupole mass spectrometer equipped with an ESI source (Agilent Technologies, Santa Clara, CA, USA) that operated in both positive ionization and negative ionization mode. The nebulizer pressure was set to 45 psi and the flow rate of drying gas was 5 L/min. The collision energy was set to 5, 15, 25 and 35 eV. The flow rate and the temperature of the sheath gas were 11 L/min and 350 °C, respectively. Chromatographic separations were done on an ODS C18 analytical column (4.6 × 250 mm) using an Agilent 1290 Infinity HPLC system (Agilent Technologies). The eluent was split and approximately 0.3 mL/min was introduced into the mass detector. An Agilent Mass Hunter Workstation was used for data acquisition and processing.

### 3.7. Antioxidant Activity Assays

DPPH radical scavenging activity was measured according to Brand-Williams *et al.* [30] with modifications. The reaction was carried out by adding sample (0.1 mL) to 60 μmol/L DPPH solution (3.9 mL) at room temperature. After 60 min, the absorbance of samples was recorded at 517 nm, by a spectrophotometer. Trolox was used as standard and data were expressed as mg TEAC/g DW.

ABTS assay was carried out using a spectrophotometer as reported [31]. ABTS radical cation was generated by reacting 7 mmol/L ABTS with 2.45 mmol/L potassium persulfate, and the mixture was allowed to stand in the dark at room temperature for 16 h before use. Before analysis, the ABTS solution was diluted with ethanol to an absorbance of 0.70 ± 0.05 at a wavelength of 734 nm. The absorbance at 734 nm was recorded for 6 min after mixing of the tested samples (0.1 mL) with ABTS solution (3.9 mL). Trolox was used as standard and data were expressed as mg TEAC/g DW.

The FRAP was measured according to Benzie *et al.* [32] with modifications. A fresh working solution was prepared by mixing 100 mL 300 mmol/L acetate buffer (pH 3.6), 10 mL 10 mmol/L TPTZ solution in 40 mmol/L HCl, and 10 mL 20 mmol/L FeCl_3_ solution. The reaction was carried out by adding sample (0.1 mL) to the FRAP solution (0.9 mL) for 10 min at 37 °C, and absorbance at 593 nm was recorded. Trolox was used as standard and data were expressed as mg TEAC/g DW. For each of the antioxidant method, an antioxidant index score was calculated according to the formula: Antioxidant index score = [(sample score/best score) × 100], and APC Index was calculated as the average of the antioxidant index score of each method.

### 3.8. Statistical Analysis

Besides the fruit quality index in Table 1, which was measured for 12 fruits for each cultivar, all the other data were the result of at least three replications and were expressed as the mean ± standard deviation. Statistical analysis was performed using SPSS 17.0 software (SPSS Inc., Chicago, IL, USA) and significant differences among the samples were calculated using one-way ANOVA followed by Duncan’s multiple range test at *p* < 0.05. Pearson correlation coefficients were calculated between antioxidant activity and phenolic contents at *p* < 0.05.

## 4. Conclusions

In the present study, the phenolic compounds and antioxidant capacities of the peel and pulp of seven loquat cultivars grown in China were investigated. Eleven phenolic compounds were identified and quantified. 3-CQA and 5-CQA were the predominant components in both tissues. Peel contained higher amounts of phenolics than pulp, and flavonols were mainly detected in the peel. In addition, the APC index of different cultivars varied from 64.15 (JJ) to 100 (DHP) in the peel and 59.49 (BZ) to 97.95 (LYQ) in the pulp. Both DHP and LYQ are the main loquat cultivars in the market, and the high APC values for both these two cultivars indicated that they may also have better health promoting values than other cultivars. Correlation analysis showed that loquat cultivars rich in HCAs showed higher antioxidant activities. Thus, these findings may provide useful information for future study and utilization of the loquat germplasm in China.
